# Snakebite incidence in two townships in Mandalay Division, Myanmar

**DOI:** 10.1371/journal.pntd.0006643

**Published:** 2018-07-09

**Authors:** Mohammad Afzal Mahmood, Dale Halliday, Robert Cumming, Khin-Thida Thwin, Mya Myint Zu Kyaw, Julian White, Sam Alfred, David Warrell, David Bacon, Win Naing, Myat Myat Thein, Nyein Nyein Chit, Sarah Serhal, Chen Au Peh

**Affiliations:** 1 School of Public Health, University of Adelaide, Adelaide, Australia; 2 School of Public Health, University of Sydney, Sydney, Australia; 3 Ministry of Health & Sport, Myanmar, University of Medicine 1 & Yangon Specialist Hospital, Yangon, Myanmar; 4 Myanmar Australia Snakebite Project, Mandalay, Myanmar; 5 Toxinology Department, Women’s & Children Hospital, Adelaide, Australia; 6 Emergency Department, Royal Adelaide Hospital, Adelaide, Australia; 7 Nuffield Department of Clinical Medicine, University of Oxford, Oxford, United Kingdom; 8 Ministry of Health, Nyi Pyi Taw, Yangon; 9 Mandalay Regional Department of Public Health, Mandalay, Myanmar; 10 Department of Renal Medicine, Royal Adelaide Hospital, Adelaide, Australia; College of Health Sciences, Bayero University Kano, NIGERIA

## Abstract

**Introduction:**

The global incidence of snakebite is estimated at more than 2.5 million cases annually, with greater than 100,000 deaths. Historically, Myanmar has one of the highest incidences of venomous snakebites. In order to improve the health outcomes of snakebite patients in Myanmar, access to accurate snakebite incidence data is crucial. The last population-based study in Myanmar was conducted more than a decade ago. In 2014, the Ministry of Health and Sports data from health facilities indicated an incidence of about 29.5 bites/ 100,000 population/year (a total of 15,079 bites). Since data from health facilities lack information about those who do not seek health care from government health services, a new population-based survey was conducted in 2 rural areas of Mandalay region. The survey data were compared to those obtained from healthcare services.

**Method:**

4,276 rural respondents in Kyaukse and Madaya townships in Mandalay Division were recruited using cluster sampling that involved random selection of 150 villages and random sampling of 30 households from each village. One adult member of each household was interviewed using a structured questionnaire.

**Results:**

One respondent from each of 4,276 households represented 19,877 residents from 144 villages. 24 people in these households had suffered snakebite during the last one year giving an annual incidence of 116/100,000. During the last ten years, 252 people suffered snakebites. 44.1% of the victims were women. 14% of the villages reported 4 or more bites during the last ten years, whereas 27% villages reported no snakebites. 92.4% of the victims recovered fully, 5.4% died, and 2% suffered long term health issues. One victim was reported to have died from causes unrelated to the snakebite. While there was no statistically significant difference between outcomes for children and adults, 4 of 38 of those under 18 years of age died compared to 7 of 133 adults between 19 to 40 years of age.

**Conclusion:**

This incidence reported by the community members points to substantially more snakebites than the number of snakebite patients attending health facilities. This higher incidence points to the need for a nation-wide population-based survey, community education about gaining access to care where antivenom is available, and to the potential need for a larger supply of antivenom and expansion of medical care in rural areas.

## Author summary

Snakebite is a major health issue affecting large numbers of people, particularly in tropical developing countries. Myanmar has one of the highest incidences of venomous snakebite in the world. Considering changes in demography, development, agricultural practices, knowledge about prevention and preventive practices, regular and accurate assessment of incidence is needed in order to improve public health programs and health services provision to improve health outcomes for snakebite patients. For that purpose, we conducted a large population based survey in Mandalay, which is one of the seven high incidence regions in Myanmar. The survey indicated a substantially higher incidence of snakebites than that suggested by the number of snakebite victims attending health care centres and hospitals. This higher incidence of snakebite has implications for community health service planning, scale of production of antivenom, and the need to improve access to health care centres or hospitals where antivenom is available, and suggests a need for community health education regarding appropriate 1^st^ aid.

## Introduction

Snakebite is a major global health issue. The estimated global annual incidence is about 5 million snakebites resulting in between 81,000 and 138,000 deaths [1]. In 1998, Chippaux [2] estimated the incidence as high as 5.4 million bites and in 2000, White [3] suggested more than 150,000 deaths. In India alone, a well-designed community-based study documented more than 45,000 deaths in 2005 [4]. Many venomous snakebites lead to severe and persisting morbidity resulting from amputation, infection, scarring, stigmatisation and psychological trauma, all resulting in substantial physical, psychological and economic disabilities and hardship. The global burden of snakebite is predominantly in tropical developing countries where manual farming practices predominate. With inadequate access to appropriate health care and the high indirect cost associated with receiving care in hospitals in major urban centres, snakebites victims and their families often face significant economic loss [5, 6].

Many snakebite victims do not seek or receive care in the formal health care sector. Epidemiological studies dependent on data from the health care system will therefore fail to capture information about these victims. This results in serious underestimation of the true snakebite incidence. This problem is exacerbated by the fact that snakebites mainly affect people in countries where health systems are evolving and the health management information systems are insufficiently robust to capture information even about those presenting to health facilities.

Global burden estimates indicate that South and Southeast Asia and sub-Saharan Africa have the highest snakebite burden in the world [1]. Historically, within Southeast Asia, Myanmar has one of the highest incidences of venomous snakebite. In 2014, Ministry of Health and Sports (MOHS) data from health facilities indicated about 29.5 bites / 100,000 population / year (a total of 15,079 bites). The most recent community level data available on snakebite in Myanmar were obtained more than a decade ago [7]. For planning an effective response to this important public health issue and to facilitate effective clinical care for all snakebite victims, it is important to re-assess the population-based snakebite incidence in addition to having information about the patients who receive care within the health system.

With demographic changes, changes to agricultural practices, education, economic development and associated changes in prevention and access to health care, it is important periodically to re-assess the incidence, morbidity, mortality, community knowledge and practices in relation to prevention and service utilisation. We conducted a survey in two townships near Mandalay to describe snakebite incidence and mortality in rural areas, as an overwhelming majority of snakebites occur in these areas. A secondary aim is to compare these survey data with data from health centre and hospital presentations. The research was conducted with ethics approval from the Ethics Committee, Department of Medical Research, Ministry of Health, Myanmar, and the Human Research Ethics Committee at the University of Adelaide.

## Methods

A survey to assess the population-based snakebite incidence in the Mandalay region was conducted as part of a large health system and community development collaborative project involving the Myanmar Ministries of Health and Industry in conjunction with the University of Adelaide and other Australian and international institutions. This collaborative project aims to improve outcomes of snakebite patients, with a focus on improving the quantity and quality of nationally produced antivenom, its distribution in quantities sufficient for the needs of the various regions of the country, health services development through in-service training and resource improvement, and community education targeting training for prevention, first aid and appropriate use of health services. As part of the larger project, this survey was conducted to measure incidence of snakebite in the previous year, snakebite mortality in the previous 10 years, and snakebite-related knowledge and practices among community members.

After consultations with Myanmar colleagues and visits to numerous candidate townships and hospitals, we determined that the appropriate combination of a high incidence of snakebite, accessibility and local support from senior health care workers could be found in the Kyaukse and Madaya Townships of Mandalay Division. The project was in collaboration with the Myanmar MOHS and had approval for these two townships only. MOHS data based on reports from all facilities across the country indicated that Mandalay region and these two townships were representative of the high incidence regions. Incidences based on MOHS data in 2014 were 29.4/100,000 across the whole country, 38.3/100,000 across the 7 highest incidence regions (where about 90% of all snakebites in the country occur), and 44.7/100,000 in Mandalay region.

In Myanmar, a ‘township’ comprises not just an urban centre, but also the surrounding rural hinterland that may cover a significant area with many rural villages. All residents of the rural areas of Kyaukse and Madaya townships in Mandalay Division were eligible for inclusion in the survey. We excluded residents who lived in urban areas of Kyaukse and Madaya Townships (16% and 9% respectively). There is a clear distinction in the Myanmar health system between urban and rural areas. The regional health management authority had separate lists for rural and urban areas and that was the basis of distinction. We excluded urban areas as we considered that snakebite would be relatively infrequent in these areas and also that the issues related to accessing treatment would be different. Data from Mandalay General Hospital (MGH) and Mandalay Teaching Hospital (MTH) provide evidence of the relative paucity of snakebite cases in urban areas compared to rural areas. MGH takes patients from the rural regions, whereas the MTH takes patients from the urban areas. In the 12 months from April 2017 till March 2018, MGH admitted a total of 856 patients, and MTH admitted only 153 patients. Also note that many rural bite victims were cared for in Township Hospitals and did not require transfer of care to MGH at any stage. Our estimate, based on the observations across the hospitals is that only about 1 out of 10 rural patients treated at the Township Hospitals required transfer to MGH. The rest were managed at their respective Township Hospitals without onward referral.

For each selected household, an adult member was interviewed. History of snakebites and snakebite-related deaths among all household residents covering a period of 10 years prior to the survey was collected.

The household was the sampling unit. The sample size was defined as 4,500 households with about 20,000 household members. This is similar to most other recent studies in South and Southeast Asia. The sample size of those studies ranged from about 2,000 to 11,000 households [8, 9, 10,11] with the exception of one large study in Sri Lanka where the sample size was 44,136 households [12].

Cluster sampling was used, in line with the method recommended in the World Health Organisation Vaccination Coverage Cluster Surveys [13]. Three stages of sampling involved: (i) stratification by township, (ii) random selection of 75 villages from each of the two townships, (iii) random sampling of 30 households from each village selected from lists of households provided by the local government health departments. Census data on the population of each village was used in stage (ii) so that random sampling of villages was done with probability proportional to size. We used a 150x30 sampling strategy, determined after considering both statistical (precision) and practical (feasibility) issues. Based on a previous survey in Myanmar (7), the anticipated snakebite incidence was 100/100,000. The design effect for snakebite incidence in Myanmar is unknown. Design effects for most variables in UNICEF’s 2009–2010 Myanmar Multiple Indicator Cluster Survey [14] (thirty households per village, as in our survey) were between 1.5 and 2 and so we used a design effect of 2 in the design of our survey. We felt that it was feasible to conduct interviews in 30 households in a single day and that 150 villages would give good geographical coverage of the study area. The resulting sample size (4,276 households, 19,877 individuals), with correction for cluster sampling methodology, was considered adequate to enable some estimation of snakebite incidence. This sample size is similar to several previous studies of snakebite incidence in Asia (8,9,10).

Data were collected using an interviewer-administered questionnaire that took about 20 minutes to complete. The structured questionnaire (Table 1) included questions about the types of snakes in the area as reported by the respondents. Photos were not shown for the respondents to identify the type of snakes sighted in the area as this method has proved misleading and unreliable. However, patients’ descriptions and use of Burmese names for the different species contributed to our tentative identifications. Snakebites during the last 10 years, first aid and health care use, snakebite outcomes, as well as knowledge and practices for prevention, first aid and treatment.

**Table 1:** Survey questions about incidence and outcome.

Question	Responses
Have any members of your household been bitten by a snake in the last 10 years?	Yes, No, Do not know
Who in the household was bitten?	Self, Other
Age of the household member at the time of bite	Age in years
When were they bitten?	Last 1 year, longer than 1 year ago but within the last ten years
What was the outcome?	Alive & fully recovered, Alive but not fully recovered, Died from snakebite, Died from causes other than snakebite
Can you tell me names of the different snakes that live here in this area?	Russell’s viper, Cobra, Krait, Green Snake, Other (Specify
During which time of the year do snakebites happen more often in this area?	Mid-Apr to Mid-June (Ta Gu to Na Yone) Mid-June to Mid-Sep (Waso-Taw Tha Linn) Mid-Sept to Mid-Dec (Tha Dinn Gyut to Nat taw) Mid-Dec to Mid-Apr (Pyah Tho to Da Baung) Do not know
In what places do most snake bites occur in this area?	Inside the house; Outside around (and close to) the house; Along a roadway/path/street; In a forest; In a field; Other (specify); Do not know
If someone from this village were bitten by a snake today, where would you first take them to get treatment, including first aid?	Sub centre, rural health centre, Station Hospital, Township Hospital, District Hospital, Private Clinic, Traditional Healer, Monastery/Pagoda, Mandalay General Hospital, Private hospital, Charity clinic–Brahamaso, Would not take them anywhere, Other (specify), Do not know
For what reasons would you decide to take the snakebite victim to the place (in Q16)?	No cost to pay the health facility, Transport cost cheaper, Better quality of health care provided, Limited by the method of transport used, Shortest time to nearest health facility, They have AV, Other (specify), Do not know

The questionnaires were administered by primary health care workers, midwives, health assistants and public health staff employed at government health centres providing outreach care. The data collectors were trained by the research team and local Project team members, all of whom were native speakers of the Myanmar language. The completed questionnaires were reviewed daily for completeness by the trained data collector supervisors and were then reviewed by the Project team field supervisors for the survey. The questionnaires were double-entered for quality assurance. The statistical analysis was conducted using SPSS (IBM SPSS Statistics Version 24) after the survey data were weighted for respondent sampling probability. Population rates and incidence were adjusted for the design effect of the survey using the SPSS Complex Samples Function. All numerical findings, including 95% confidence intervals, are adjusted for the three stage sampling methodology.

## Results

The survey was conducted in 74 villages in Kyaukse and 70 villages in Madaya townships. One respondent from each of the 4,276 households was interviewed (participation rate of 94%). The total number of people in these 4,276 households was 19,877. 49.9% of the respondents were women. 29.1% were 21–40 years of age, 43.8% were 41–60 years of age, and 20.5% were more than 60 years of age.

### Snakebite incidence

In the 4,276 study households, representing 19,877 residents, 24 people had suffered a snakebite during the previous one year, an unadjusted incidence of 123/100,000 and adjusted incidence of 116/100,000 people (95% CI 74/100000–182 /100,000).

252 respondents informed us that at least one person in their households had been bitten by a snake during the previous 10 years (5.9% of households, 95% CI 4.8%–7.3%). Among the victims, 44.1% were women. Relatively fewer households in Madaya Township had experienced a snakebite (4.7% of households, 95% CI 3.3%–6.7%) during the last 10 years than in Kyaukse Township (7.3%, 95% CI 6.7–9.2%). 111 of 252 (44%) snakebites were reported from 21 villages (14% of the villages) were there had been 4 or more snakebites during the last 10 years. From 40 villages, there were no reports of snakebites, and only 1 snakebite was reported from 38 villages during the previous 10 years, 2 snakebites in 25 villages, and 3 snakebites in 19 villages.

### Snakebite outcomes

Outcomes were reported for 230 victims in the previous 10 years. The respondents informed us that 209 (92.4%, 95% CI 87.6%–95.5%) of these had fully recovered, 14 (5.4%, 95% CI 2.9%–10.0%) died due to that snakebite, and 6 (2%, 95% CI 0.9–4.7%) did not recover fully and had long term health issues as a result of the snakebite. It was reported that one victim died from causes unrelated to the snakebite. While there was no statistically significant difference between poor outcomes for children and adults, 4 of 38 victims under 18 years of age died compared to 7 of 133 adults 19 to 40 years of age. A higher proportion of women died (7 out of 87) than of men (7 out of 141). This difference was not statistically significant.

### Snakes in the area, as reported by the respondents

The species of snakes reported are listed in Table 2.

**Table 2:** Types of snakes reported as present in the area by villagers.

Snake Type	Likely identification of the snake, represented by scientific name	Number of Respondents Who informed us that these snakes are present in the area
Russell's Viper	*Daboia siamensis*	3393 (79.3%)
Cobra	*Naja spp*.	2305 (53.9%)
Green Snake	*Trimeresurus spp*.	2136 (50.0%)
Common rat snake	*Ptyas mucosas or Coelognathus radiata*	1742 (40.7%)
Krait	*Bungarus spp*.	1340 (31.3%)
Checkered keel-back snake	*Xenochrophis piscator*	881 (20.6%)
Myat Shaw	*Natrix piscator*	477 (11.2%)
“Boa constrictor”	*Python spp*.	285 (6.7%)
Gliding snake	*Chrysopelea spp*.	214 (5.0%)
Chinese banded krait	*Bungarus wanghaotingi or magnimaculatus*	207(4.8%)
King Cobra	*Ophiophagus hannah*	133 (3.1%)
Kukri snake	*Oligodon spp*.	4 (0.1%)
Sin Pyit Mway	*Bungarus magnimaculatus*	4 (0.1%)
Mwe Pa Dote	*Homalopsis buccata*	50 (1.2%)
Nga Pyaw Mwe	*unknown*	5 (0.1%)

The respondents informed us that most snakebites occur in and around fields and in the forests (Table 3).

**Table 3:** Places around the village/house where snakebites occur more often.

Place	Frequency	
In a forest	2001	50.3%
In a field	1798	46.6%
Outside/around house	41	3.1%
Along a roadway/path	40	1.3%
Inside the house	19	0.8%
Hilly region	8	0.6%
Do not know	179	6.0%
No answer	190	5.5%
Total	4276	100.0%

Snakebites were reported to occur during all four seasons but peak incidence was believed to during hot periods before the monsoon (Apr-June–Tu Gu—Na Yone season) and during the monsoon period (Table 4). (Note that Tu Gu—Na Yone is shorter than the other three seasons.) The activities most associated with people being bitten were farming and collecting crops (Table 5).

**Table 4:** Time of the year when snakebite occurs more often.

Time of the Year	Frequency (%)
Mid April to Mid June (Ta Gu to Na Yone)	1016 (24.9%)
Mid June to Mid September (Waso-Taw to Tha Linn)	1158 (25.4%)
Mid Sept to Mid December (Tha Dinn Gyut to Nat Taw	872 (19.4%)
Mid December to Mid April (Pyah Tho to Da Baung)	666 (15.6%)
Not Known	435 (11.6%)

**Table 5:** Activities most associated with people getting bitten.

Activity	Frequency	%
Lying, resting or sleeping	5	0.1%
While in the toilet	9	0.2%
Walking home from fields	370	8.4%
Fishing	10	0.2%
Cutting wood in the forest	123	3.3%
Tending animals	67	1.5%
Farming	2205	51.5%
Collecting crops	1086	24.8%
Constructions	3	0.0%
Do not know	178	4.9%
No answer	220	5.1%
Total	4276	100.0%

Considering the likely better recall about symptoms and treatment for those 24 snakebite victims who were bitten during the one year preceding the survey, the household members were asked questions about symptoms and treatment received. 6 (25%) of those 24 that reported snakebite to one of their family members reported that it was a venomous snake, whereas 3 (12.5%) reported it was not a venomous snake. Of the 24 respondents 19 could not recall the type of treatment received. Three reported that the victim received anti-venom. There were 2 reports of intravenous fluid being used with one reported to have received oxygen as well. The respondents informed that 8 (33.3%) of these 24 patients fully recovered. Others did not report if their family member fully recovered or had long term consequences.

## Discussion

Our 2015 survey discovered an incidence of snakebite 116/100,000 in two areas chosen for their high incidence of snakebite. This is much higher than the 2014 and 2016 national incidence as reported in the public health statistics [15] and an incidence of 68.5/100,000 rural populations in Mandalay region calculated using the Ministry of Health and Sports health facilities statistics and Myanmar Information Management Unit Census [15, 16, 17].

Considering the relatively small number of snakebites as reported by the respondents pointing to an estimated incidence of 116/100,00 with wider confidence intervals, our extrapolations must be interpreted with caution. The incidence identified through our survey suggests that the number of snakebites in the rural areas of seven regions of Myanmar, where 90% of snakebites occur and where about 23 million people live, may be significantly higher than the number of snakebites reported in the national health facilities data. Nationally, in 2014, 15,079 snakebite patients sought medical care from government facilities, out of which 13,382 were from the seven high incidence regions (Mandalay, Sagaing, Bago, Magwe, Yangon, Nay Pyi Taw and Ayeryarwady). According to health facility data, the snakebite in all these high incidence regions (except Nay Pyi Taw, where incidence in about 19/100,000), range between 38/100,000 and 52/100,000, with an incidence in Mandalay of 44/100,000. This suggests that information about incidence in one of these regions might be applied to the others.

The case fatality rate of snakebite was about 5% in the rural areas of Kyaukse and Madaya where this survey was conducted. This suggests about 1,250 deaths (0.05 x 25,000) due to snakebite in the 23 million people living in the rural areas of 7 high incidence regions. The total number of deaths caused by snakebites across the whole of Myanmar as reported in the national health services data was 630 in 2014 [16]. This paper reports the community based incidence based on a community survey. As the overall incidence per 100,000 population is relatively low and for that reason despite a relatively larger sample size it was not possible to explore the health services use and outcomes in the community survey. Therefore, to further understand the health services use and health outcomes for a large number of patients, a hospital record database was established at Mandalay General Hospital (MGH). MGH admits patients from across the whole region, mainly from the rural areas. In 2016, 965 patients were admitted to that hospital. A minority, 11.5%, sought care from traditional healers before visiting a formal health care facility, while 87.7% sought care from the formal health system as their first point of contact. A large majority, 85.4%, of patients were bitten by Russell’s vipers (RV), and all 9.8% deaths of patients admitted to this hospital were caused by RV. The complications included Acute Kidney Injury, (59.8%), requiring dialysis in 23.9% cases. Green pit viper bites were the next most common cause of bites (7.6%).

The reasons why relatively fewer snakebites and snakebite deaths were recorded in the health services data may include failure of some victims to seek health care, or seeking care from traditional healers only, and deaths of some victims before they could seek medical care. A proportion of snakebites cause no symptoms of envenoming: because the venomous snake injects no venom (“dry bites”), because some bites are by non-venomous snakes or other animals, or because the “bite” was just a sharp injury from a thorn. These victims may decide not to seek medical care because they develop no symptoms.

Among other factors, characteristics of a disease under investigation influence recall bias [18]. It could be assumed that there is a good recall for snakebite compared to other diseases, because snakebite is so memorable and is greatly feared as it is life threatening and causes serious complications including amputations and deaths in many patients. The concordance of our survey’s one year (24 bites) and ten year (252 bites) incidences suggests that the information provided by the respondents is affected little by recall bias. Hence, we are confident that the incidence identified by our survey is close to the true incidence in our study area. A study in 2005 in two other townships of the high incidence regions of Myanmar reported similarly high annual incidence of as high as 100/100000 in Kyaukapadaung and 115/100000 in Taungdwingyi Township [7]. Myanmar is in a region having one of the highest snakebite incidence in the world. Kasturiratne reported that South and South East Asia suffer about 232,000 envenomings every year [19]. A survey in Bangladesh reported an incidence of 623/100,000 [8] and a survey in Laos reported an incidence of 355/100,000 in one area and 1162/100000 in another [9].

The high snakebite incidence and case fatality rate identified through this survey has important implications. There is an imperative to work with the communities to raise awareness about the need for early access to health care facilities by all of the victims regardless of whether envenoming has already occurred or not. While it is true that a proportion of bites by venomous snakes do not cause envenoming, to avoid delays in treatment leading to severe envenoming and systemic complications it is important that the victims are taken, as soon as possible after the bite, to health care facilities where antivenom is available. There, they should be observed and tested for symptoms and signs of envenoming so that treatment with relevant antivenom can be provided if needed. The long term complications of snakebites from Russell’s Vipers, which are responsible for most of the bites in this region, have not been studied formally. However, long term complications seen among the snakebite patients across the project hospitals including Mandalay General hospital include chronic renal failure, chronically infected bite site wound, hypopituitarism and loss of a limb from amputation. This aspect of snakebite sequelae requires further research in the future.

Our findings suggest a higher incidence of snakebite than recorded in health services data. This has implications for service planning, including the potential need for production of more antivenom.

The survey also revealed the large variety of snakes known to be present in this area. Symptoms of envenoming and envenoming syndromes, i.e. haemotoxic, neurotoxic, nephrotoxic, help make a more precise determination of snake species. However, as it would have been difficult for the community members to remember the symptoms correctly, this survey, which focussed on incidence, did not include questions about signs and symptoms affecting those who were reported bitten in the last ten years. Other aspects of the project, particularly examining of dead snakes brought by the patients to Mandalay General Hosital, confirmed that most of the snakebites in this area are by Russell’s Viper, Rat Snake, Cobra, Checkered Keelback and Green Snakes. A number of the snakes reported by the respondents are non-venomous. Some of the victims may not seek care if they consider that have been bitten by a non-venomous snake if envenoming symptoms do not appear early. There is a need for a herpetological survey in high snakebite incidence regions of the country.

Another important finding is that even within an overall high incidence region most of the snakebites may be clustered in some areas, with other areas having relatively few or no snakebites. Out of 144 villages, respondents from 40 villages reported no snakebite during the previous ten years. Likely reasons, that require further exploration, include the type of crops grown, proximity to rivers and marshy areas, sanitation in and around houses, and use of personal protective equipment such as long boots. This heterogeneous incidence of bites demands that the trends and situation within an area be carefully assessed when planning targeted health education, antivenom distribution, and in-service training for the health care providers and managers who work in the high incidence areas.

This survey was one of the many aspects of the health system development project. Other initiatives included auditing of hospital patient data, participatory research with the communities, examination of dead snakes brought by the patients, and discussions with the primary care staff about the snakebite problem in the area. These initiatives helped generate sufficient information to be able to assist the government health services in strengthening the algorithms for diagnosis and treatment, training a large number of staff at primary care facilities and hospitals, greatly increasing the quantity of antivenom, and lyophilisation of antivenom to avoid difficulties associated with cold chain in areas lacking electricity networks.

### Limitations

We acknowledge that the sample size was too small to provide a precise estimate of snakebite incidence. With only 24 snakebites in the past year, the point estimate of 116 snakebites per 100,000 population had wide confidence intervals (74/100,000 to 182/100000). To achieve a precise estimate of snakebite incidence in Myanmar would require a sample size of at least 100,000 people.

The lack of detailed information about symptoms, treatment and outcomes including long term consequences is another limitation. Considering difficulty in recalling clinical information by the community members it is challenging to capture such clinical information through a community survey. Research that involves reviewing the data of patients admitted to the township, district and referral hospitals will help capture such clinical information which when used in conjunction with the information about incidence would help refine and strengthen in-service trainings and better define which anti venoms are necessary.

## Supporting information

S1 Checklist STROBE checklist. Checklist of items for this observational study.
(PDF)
