# Supplementary material for: Snakebite incidence in two townships in Mandalay Division, Myanmar
Source: PLoS Negl Trop Dis. 2018 Jul 9;12(7):e0006643. doi: 10.1371/journal.pntd.0006643 (PMC6053239; doi:10.1371/journal.pntd.0006643)
Supplement: S1 Checklist — Checklist of items for this observational study. (PDF) [file pntd.0006643.s001.pdf]

STROBE Statement for ***cross-sectional studies***

Snakebite Incidence in Two Townships in Mandalay Division, Myanmar

	Item No	Recommendation
**Title and abstract**	1	(*a*) Line 2
		(*b*) Lines 30-54
**Introduction**		
Background/rationale	2	Lines 72-94
Objectives	3	Lines 96-103
**Methods**		
Study design	4	Lines 106-107, 117-125
Setting	5	Lines 117-121
Participants	6	Lines 121-125
Variables	7	Lines 124-125, 139-142, 146-148
Data sources/ measurement	8*	Lines 127-128
Bias	9	Lines 132-137, 150-154
Study size	10	Lines 127-130
Quantitative variables	11	Lines 154-157
Statistical methods	12	Lines 154-157
		Analytical method for sampling strategy – line 155
		Sub-groups and interactions – Not Applicable
		Sensitivity Analysis – Not Applicable
**Results**		
Participants	13*	Lines 160-161
Descriptive data	14*	Lines 162-163
Outcome data	15*	NA
Main results	16	Lines 165-204
Other analyses	17	NA
**Discussion**		
Key results	18	Lines 208-216, 218-221
Limitations	19	Lines 263-266
Interpretation	20	Lines 223-228, 230-238, 240-246
Generalisability	21	Lines 219-220
**Other information**		
Funding	22	DFAT, Australia. Information provided in online submission system

*Give information separately for exposed and unexposed groups.

Note: An Explanation and Elaboration article discusses each checklist item and gives methodological background and published examples of transparent reporting. The STROBE checklist is best used in conjunction with this article (freely available on the Web sites of PLoS Medicine at http://www.plosmedicine.org/, Annals of Internal Medicine at http://www.annals.org/, and Epidemiology at http://www.epidem.com/). Information on the STROBE Initiative is available at www.strobe-statement.org.
